# A PAS-targeting hERG1 activator reduces arrhythmic events in Jervell and Lange-Nielsen syndrome patient-derived hiPSC-CMs

**DOI:** 10.1172/jci.insight.183444

**Published:** 2025-01-09

**Authors:** Chiamaka U. Ukachukwu, Eric N. Jimenez-Vazquez, Shreya Salwi, Matthew Goodrich, Francisco G. Sanchez-Conde, Kate M. Orland, Abhilasha Jain, Lee L. Eckhardt, Timothy J. Kamp, David K. Jones

**Affiliations:** 1 Department of Pharmacology, University of Michigan Medical School, Ann Arbor, Michigan, USA.; 2 Cellular and Molecular Arrhythmia Research Program, Division of Cardiovascular Medicine, Department of Medicine, University of Wisconsin-Madison, Madison, Wisconsin, USA.; 3 Inherited Arrhythmia Clinic, Division of Cardiovascular Medicine, Department of Medicine, Madison, Wisconsin, USA.; 4 Department of Cell and Regenerative Biology, University of Wisconsin-Madison, Madison Wisconsin, USA.; 5 Department of Internal Medicine, University of Michigan Medical School, Ann Arbor, Michigan, USA.

**Keywords:** Cardiology, Arrhythmias, Ion channels, Potassium channels

## Abstract

The hERG1 potassium channel conducts the cardiac repolarizing current, I_Kr_. hERG1 has emerged as a therapeutic target for cardiac diseases marked by prolonged action potential duration (APD). Unfortunately, many hERG1 activators display off-target and proarrhythmic effects that limit their therapeutic potential. A Per-Arnt-Sim (PAS) domain in the hERG1 N-terminus reduces I_Kr_ by slowing channel activation and promoting inactivation. Disrupting PAS activity increases I_Kr_ and shortens APD in human induced pluripotent stem cell–derived cardiomyocytes (hiPSC-CMs). We thus hypothesized that the hERG1 PAS domain could represent a therapeutic target to reduce arrhythmogenic potential in a long QT syndrome (LQTS) background. To test this, we measured the antiarrhythmic capacity of a PAS-disabling single-chain variable fragment antibody, scFv2.10, in a hiPSC-CM line derived from a patient with Jervell and Lange Nielsen (JLN) syndrome. JLN is a severe form of LQTS caused by autosomal recessive mutations in *KCNQ1*. The patient in this study carried compound heterozygous mutations in *KCNQ1*. Corresponding JLN hiPSC-CMs displayed prolonged APD and early afterdepolarizations (EADs). Disrupting PAS with scFv2.10 increased I_Kr_, shortened APD, and reduced the incidence of EADs. These data demonstrate that the hERG1 PAS domain could serve as a therapeutic target to treat disorders of cardiac electrical dysfunction.

## Abstract 2

Disrupting a cytoplasmic domain of the cardiac potassium channel hERG1 reduces markers of proarrhythmia in a model of long QT syndrome.

## Introduction

*KCNH2* encodes the voltage-gated potassium channel (hERG1/Kv11.1) that conducts the rapid delayed rectifier potassium current, I_Kr_ (1, 2). hERG1 activation is a promising avenue to treat diseases of electrical excitability (3–6), yet current small-molecule hERG1 agonists lack sufficient specificity and were shown to increase arrhythmogenesis despite increasing I_Kr_ (3, 7). Thus, identifying novel targets to increase I_Kr_ may circumvent the current limitations of hERG1 agonists to treat cardiac electrical dysfunction.

Native cardiac hERG1 channels are composed of at least 2 subunits, hERG1a and hERG1b, that are identical except for their N-termini (8–12). The hERG1a N-terminal domain contains a Per-Arnt-Sim (PAS) domain that regulates channel gating through a direct interaction with its C-terminal cyclic nucleotide binding homology domain (CNBHD) and the cytoplasmic S4-S5 linker (13–15). hERG1b has a unique and shorter N-terminus that lacks a PAS domain (10, 11). Channels containing hERG1b display faster deactivation, activation, and inactivation recovery compared with homomeric hERG1a channels (9, 16). Consequently, heteromeric hERG1a/1b channels conduct roughly twice as much current as homomeric hERG1a channels (9, 17). Conversely, overexpressing a polypeptide identical to the hERG1a PAS domain slows heteromeric hERG1a/1b channel gating to a phenotype identical to homomeric hERG1a-like channels (12, 18). In healthy human induced pluripotent stem cell–derived cardiomyocytes (hiPSC-CMs), PAS transduction reduces I_Kr_ magnitude and prolongs action potential duration (APD) compared with GFP controls (12). These data suggest that the hERG1a PAS domain acts to suppress I_Kr_ in human cardiomyocytes and could be targeted to enhance I_Kr_ in cases of impaired cardiac repolarization.

Previous works identify a single-chain variable fragment antibody, scFv2.10, a novel hERG1 activator that selectively binds the hERG1 PAS domain and disrupts the PAS-CNBHD interaction (19, 20). In HEK293 cells stably expressing hERG1a, intracellular delivery of purified scFv2.10 accelerated the time course of deactivation, slowed the onset of inactivation, and increased hERG1 current magnitude (19). In hiPSC-CMs, intracellular scFv2.10 delivery increased I_Kr_ magnitude and shortened APD (19). Based on these findings, we sought to use scFv2.10 to test the hypothesis that the hERG1a PAS domain represents a novel antiarrhythmic drug target to treat disorders of impaired cardiac repolarization.

We tested the antiarrhythmic capacity of disrupting PAS action in a hiPSC-CM line derived from a patient with Jervell and Lange-Nielsen (JLN) syndrome. JLN is a severe form of long QT syndrome (LQTS), caused by homozygous or compound heterozygous mutations in KCNQ1 or KCNE1, creating functional KOs of the slowed delayed rectifier potassium current, I_Ks_ (21–23). Patients with JLN are characterized by QT prolongation, syncope, congenital deafness, and increased risk for cardiac arrhythmia and sudden cardiac death (24). In our study, JLN hiPSC-CMs displayed prolonged action potentials, as well as increased action potential variability and incidence of early afterdepolarizations (EADs) compared with hiPSC-CMs derived from a healthy patient background. Disrupting PAS activity in JLN hiPSC-CMs by overexpressing scFv2.10 increased I_Kr_, shortened the APD, and reduced both action potential variability and the incidence of EADs compared with GFP-transduced JLN hiPSC-CMs. These data demonstrate that disabling the PAS domain may be a viable strategy to selectively enhance hERG1 current and treat diseases of disrupted cardiac repolarization.

## Results

### hiPSC line generation from patients with JLN.

**Figure 1:**
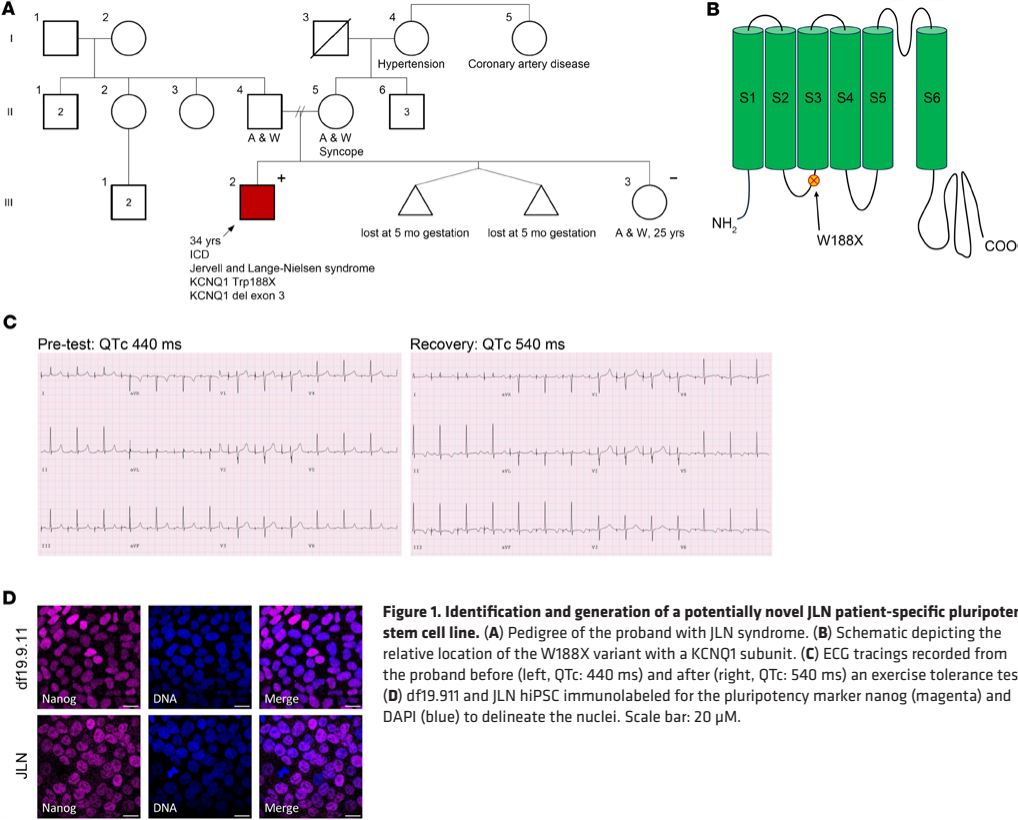
Identification and generation of a potentially novel JLN patient-specific pluripotent stem cell line. (**A**) Pedigree of the proband with JLN syndrome. (**B**) Schematic depicting the relative location of the W188X variant with a KCNQ1 subunit. (**C**) ECG tracings recorded from the proband before (left, QTc: 440 ms) and after (right, QTc: 540 ms) an exercise tolerance test. (**D**) df19.911 and JLN hiPSC immunolabeled for the pluripotency marker nanog (magenta) and DAPI (blue) to delineate the nuclei. Scale bar: 20 µM.

We generated a novel patient-derived hiPSC line from a patient with JLN who suffered congenital deafness, syncopal events, and seizures and was diagnosed with LQTS in childhood. As a child, he had syncope with loss of consciousness, and an ECG demonstrated QT prolongation. He was treated with metoprolol but continued to have syncope; thus, at age 10, the patient was given a dual-chamber implantable cardioverter defibrillator (ICD). His initial research-related genotyping identified c.563G>A,Trp188X in *KCNQ1*, a nonsense variant causing premature termination in the S2-S3 linker (Figure 1, A and B). The patient was prescribed metoprolol and suffered recurrent syncope and appropriate shocks as a teenager. At age 18, the patient was seen at the University of Wisconsin Inherited Arrhythmias Clinic and switched to nadolol, 80 mg (25), and has been event free since. An exercise-tolerance test on nadolol 1 year later demonstrated excessive QT prolongation (540 ms) more than 4 minutes into recovery, consistent with a loss-of-function *KCNQ1* variant (Figure 1C). Due to the suspicion for JLN, additional sequence analysis at that time (PCR based) showed apparent homozygosity with Trp188X on both chromosomes, a phenomenon only possible with consanguinity. Knowing that consanguinity was not present, the Inherited Arrhythmias Clinic pursued Multiplex Ligation-Dependent Probe Amplification (MLPA) and identified a deletion of exon 3 in the second *KCNQ1* allele (Figure 1B, ΔExon 3). We generated JLN patient-specific iPSCs as previously described (26). Sequence analysis of genomic DNA verified that iPSC clones from the patient with JLN carried the c.563G>A/ΔExon 3 complex variant in *KCNQ1* (NM_000218.3) (Figure 1, A and B). Given the complexity of the *KCNQ1* variants, it was not feasible to generate an isogenic control. We instead used the healthy control hiPSC line, df19.9.11, provided by WiCell (26). Both JLN and df19.9.11 stem cell lines displayed appropriate morphologies and markers of pluripotency (Figure 1D).

### JLN hiPSC-CMs display markers of proarrhythmia.

**Figure 2:**
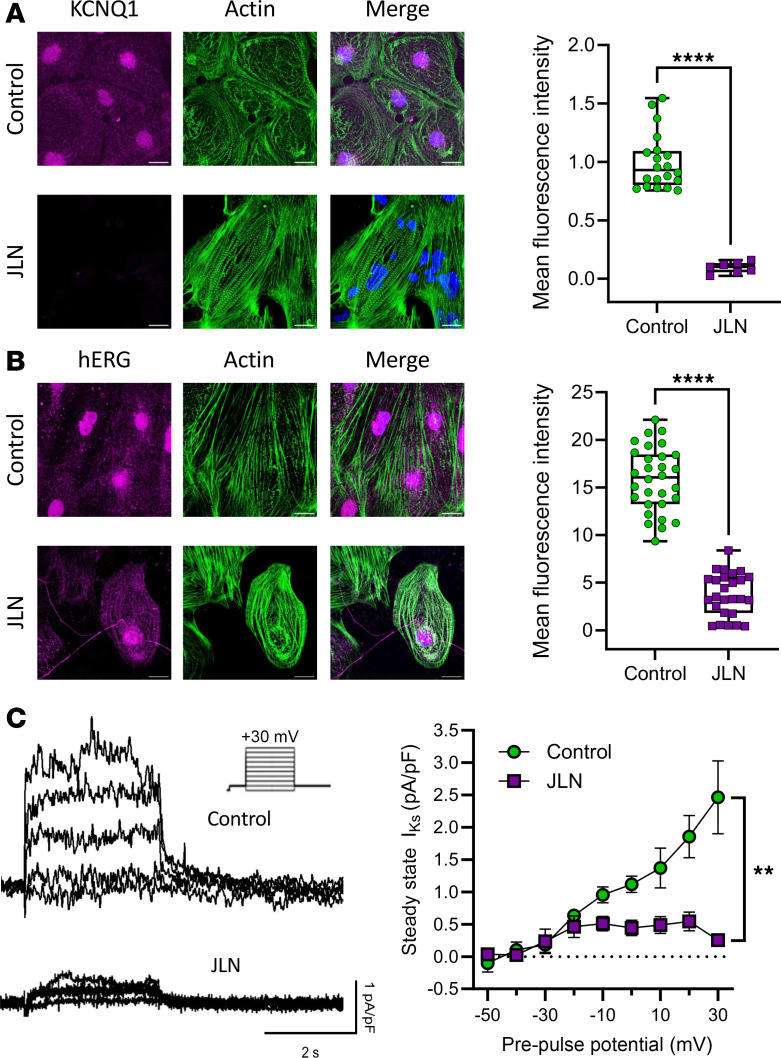
Validation of JLN-derived hiPSC-CMs. (**A**) Sample max intensity images of df19.9.11 (Control, top) and JLN (bottom) hiPSC-CMs depicting KCNQ1 (magenta), phalloidin (green), and DAPI (blue). Mean fluorescence is quantified (right). (**B**) Sample max intensity images of df19.911 (top) and JLN (bottom) hiPSC-CMs depicting hERG1 (magenta), phalloidin (green), and DAPI (blue). Mean fluorescence intensity quantified (right). (**C**) Sample I_Ks_ traces recorded from df19.9.11 (top) and JLN (bottom) hiPSC-CMs. Steady-state I_Ks_ density plotted as a function of prepulse potential for df19.911 (green) and JLN (magenta) hiPSC-CMs. *P* values were determined by unpaired, 2-tailed Student’s *t* test, or ordinary 2-way ANOVA (mixed methods) with multiple comparisons and Šidák post hoc test. **P < 0.01, ****P < 0.0001. Data are presented as mean ± SEM. Scale bar: 20 μm.

**Figure 3:**
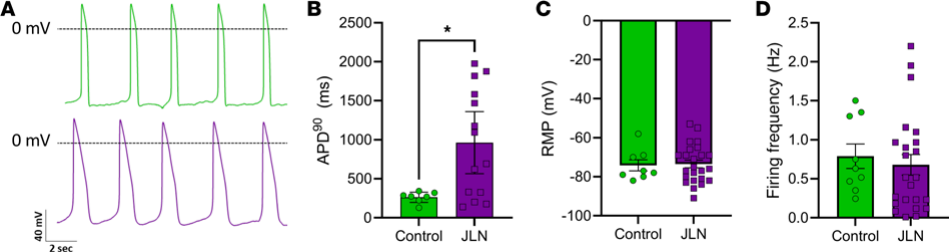
JLN hiPSC-CMs display prolonged APD_90_. (**A**) Representative traces of spontaneous action potentials recorded from df19.911 (green) and JLN (purple) hiPSC-CMs. (**B**–**D**) APD_90_ (**B**), resting membrane potential (**C**), and spontaneous firing frequency (**D**) recorded from df19.911 and JLN hiPSC-CMs. *P* values were determined by unpaired, 2-tailed Student’s *t* test. RMP, resting membrane potential. *P < 0.05. Data are presented as mean ± SEM.

We differentiated hiPSCs into ventricular-like cardiomyocytes using the GiWi protocol, as described (27). To verify the appropriate molecular and electrophysiological phenotype of differentiated JLN hiPSC-CMs, we measured KCNQ1/K_V_7.1 and hERG1 channel immunofluorescence, chromanol-293b–sensitive currents indicative of I_Ks_ (Figure 2), and spontaneous action potentials from healthy control (df19.9.11, WiCell) and JLN hiPSC-CMs (Figure 3 and Table 1). df19.911 hiPSC-CMs displayed robust KCNQ1 and hERG1 immunofluorescence (Figure 2, A and B) and conducted a voltage-dependent chromanol-293b sensitive current (Figure 2C). In contrast, JLN hiPSC-CMs displayed effectively no KCNQ1 immunofluorescence or chromanol-293b–sensitive current (Figure 2, A and C). Surprisingly, we identified a nuclear KCNQ1 immunofluorescence signal in healthy control hiPSC-CMs, that was absent in JLN hiPSC-CMs (Figure 2A). The absence of the KCNQ1 immunofluorescence signal in the JLN hiPSC-CMs validates the nuclear KCNQ1 signal. To our knowledge, this is the first report of a nuclear KCNQ1 immunofluorescence signal, whereas hERG1-specific nuclear immunofluorescence has been reported previously (27, 28). Notably, hERG1 expression in JLN hiPSC-CMs was 4-fold less than in df19.911 hiPSC-CMs (Figure 2B, right). Spontaneous action potentials recorded from JLN hiPSC-CMs were substantially prolonged (90% of APD [APD_90_], 963 ± 184 ms) compared with df19.911 hiPSC-CMs (APD_90_, 263 ± 27 ms) (Figure 3, A and B) but displayed no significant differences in resting membrane potential or spontaneous firing frequency (Figure 3, C and D). These data confirm key proarrhythmic features of the JLN-derived hiPSC-CMs and are in agreement with previous work showing that KCNQ1 disruption reduces hERG1 expression (29).

### scFv2.10 overexpression increases I_Kr_ magnitude in JLN hiPSC-CMs.

**Figure 4:**
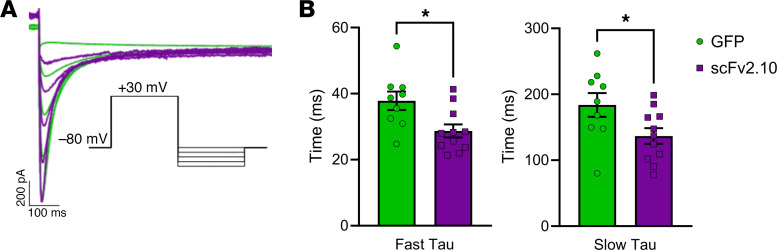
scFv2.10 transduction accelerates gating in HEK293 cells stably expressing hERG1a. (**A**) Representative deactivation traces from GFP-transduced (green) or scFv2.10-transduced (purple) HEK 293 cells. Pulse protocol shown in inset. (**B**) Fast and slow time constants of deactivation measured at –110 mV. Data are presented as mean ± SEM. *P* values were determined by unpaired, 2-tailed Student’s *t* test. *P < 0.05. Data are presented as mean ± SEM.

The time course of deactivation is a reliable proxy of PAS activity in hERG1a channels, where accelerated deactivation correlates with decreased or impaired PAS activity (9, 16, 18). Accordingly, previous work demonstrated that purified scFv2.10 delivered through the recording pipette accelerates hERG1a deactivation (19, 30). To confirm that lentiviral scFv2.10 expression can generate sufficient levels of functional scFv2.10 polypeptide to modulate hERG1a gating, we measured hERG1a current deactivation from HEK293 cells stably expressing hERG1a transduced with either scFv2.10 or GFP. hERG1a deactivation recorded from scFv2.10-transduced cells was significantly accelerated compared with GFP-transduced controls (Figure 4), indicating that virally expressed scFv2.10 was functional.

**Figure 5:**
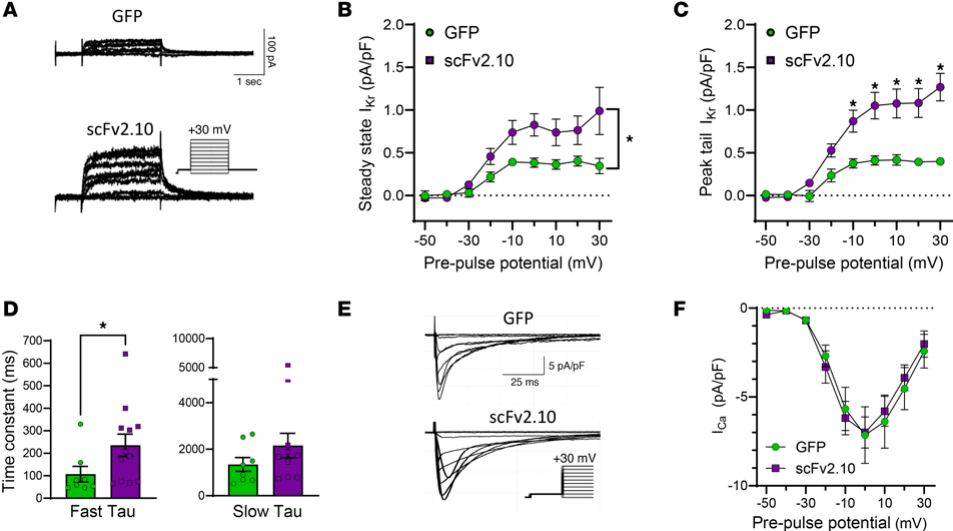
scFv2.10 transduction selectively increases I_Kr_ density in JLN hiPSC-CMs. (**A**) Representative I_Kr_ traces from JLN hiPSC-CMs transduced with GFP or scFv2.10 elicited by the pulse protocol shown in the inset. (**B**) Steady-state I_Kr_ recorded from GFP-transduced (green circles) and scFv2.10-transduced (magenta squares) hiPSC-CMs plotted as a function of prepulse potential. (**C**) Peak tail I_Kr_ recorded from GFP-transduced (green circles) and scFv2.10-transduced (magenta squares) hiPSC-CMs plotted as a function of prepulse potential. (**D**) Fast and slow deactivation time constants recorded from JLN hiPSC-CMs at +20 mV. (**E**) Representative I_Ca_ traces from JLN hiPSC-CMs transduced with GFP or scFv2.10. (**F**) I_Ca_ plotted as a function of prepulse potential. *P* values were determined by unpaired, 2-tailed Student’s *t* test, or ordinary 2-way ANOVA (mixed methods) with multiple comparisons and Šidák post hoc test. *P < 0.05. Steady-state and Peak tail I_Kr_ *n* values (*N* = 2) are 7 and 10 for GFP and scFv2.10 transduced hiPSC-CMs, respectively. I_Ca_ *n* value (*N* = 2) is 10 for GFP and 9 for scFv2.10 transduced hiPSC-CMs. Data are presented as mean ± SEM.

To determine the antiarrhythmic capacity of disabling the hERG1a PAS domain, we transduced JLN hiPSC-CMs with the scFv2.10 polypeptide and measured its effect on cardiac currents and APs. In JLN hiPSC-CMs, scFv2.10 transduction increased steady-state and tail I_Kr_ by roughly 2-fold compared with GFP-transduced cells (Figure 5, A–C, and Table 2). Increased tail I_Kr_ is a proxy for increased channel trafficking to the membrane; thus, these data suggest that scFv2.10 increased hERG1 abundance at the surface membrane. Surprisingly, scFv2.10 transduction slowed the time course of deactivation in JLN hiPSC-CMs (Figure 5D). scFv2.10 did not affect the magnitude of voltage-dependent I_Ca_ in JLN hiPSC-CMs, suggesting that scFv2.10 is I_Kr_ selective in cardiomyocytes (Figure 5, E and F), as previously reported (19). Voltage-dependent I_Ca_ also served as an indicator of healthy excitable cardiomyocytes.

### scFv2.10 expression alters hERG1 subunit abundance in JLN hiPSC-CMs.

scFv2.10 transduction slowed the time course of I_Kr_ deactivation, suggesting that, in hiPSC-CMs, scFv2.10 transduction enhanced PAS activity. Altered hERG1a/hERG1b subunit abundance could explain this unexpected result, where elevated hERG1a subunits in the presence of scFv2.10 slows the time course of deactivation.

**Figure 6:**
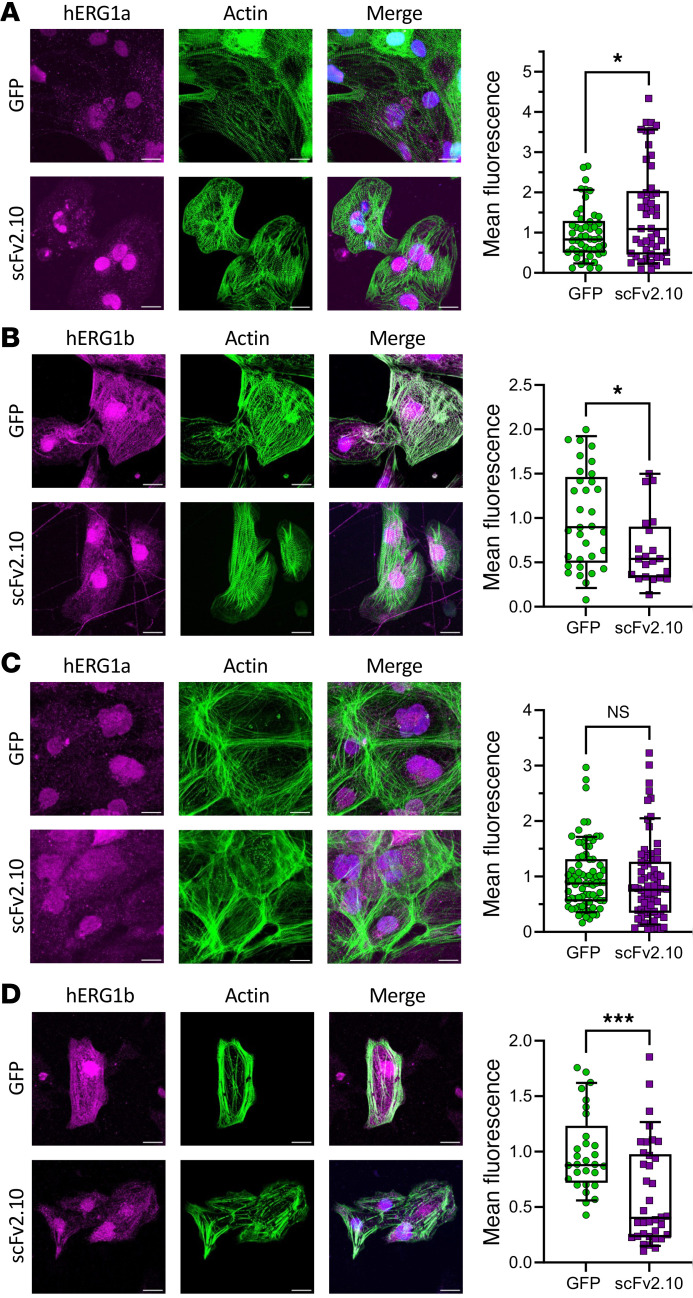
scFv2.10 transduction shifts hERG1 subunit abundance. (**A** and **B**) Sample max intensity images of JLN hiPSC-CMs depicting hERG1a/1b (magenta), phalloidin (green), and DAPI (blue). scFv2.10 transduction significantly upregulated hERG1a (**A**) and downregulated hERG1b (**B**) compared with GFP controls. (**C** and **D**) Sample max intensity images of df19.9.11 hiPSC-CMs depicting hERG1a/1b (magenta), phalloidin (green), and DAPI (blue). scFv2.10 transduction of df19.9.11 hiPSC-CMs did not affect hERG1a immunofluorescence (**C**) but downregulated hERG1b immunofluorescence (**D**) compared with GFP controls. *P* values were determined by unpaired, 2-tailed Student’s *t* test. *P < 0.05, ***P < 0.001. Scale bar: 20 μm. Data are presented as median ± 95% CI.

To test if scFv2.10 transduction alters hERG1 subunit abundance, we measured hERG1a-specific and hERG1b-specific immunofluorescence in JLN hiPSC-CMs transduced with either scFv2.10 or GFP (Figure 6). Consistent with previous work, hERG1a and hERG1b displayed both membrane-targeted and nuclear fluorescence (28). hERG1a immunofluorescence was increased in scFv2.10-transduced JLN hiPSCCMs compared with GFP-transduced controls (Figure 6A). Conversely, hERG1b immunofluorescence was decreased in scFv2.10-transduced JLN hiPSCCMs compared with GFP-transduced controls (Figure 6B). These data demonstrate that lentiviral expression of scFv2.10 promotes hERG1a abundance relative to hERG1b and may explain the slowing of I_Kr_ deactivation in the presence of scFv2.10. In WT df19.9.11 cells, hERG1b immunofluorescence was also significantly reduced (Figure 6D), whereas hERG1a immunofluorescence was unchanged (Figure 6C). These data suggest that scFv2.10 transduction alters hERG1 subunit dynamics in hiPSC-CMs.

### scFv2.10 reduces arrhythmic events in JLN hiPSC-CMs.

**Figure 7:**
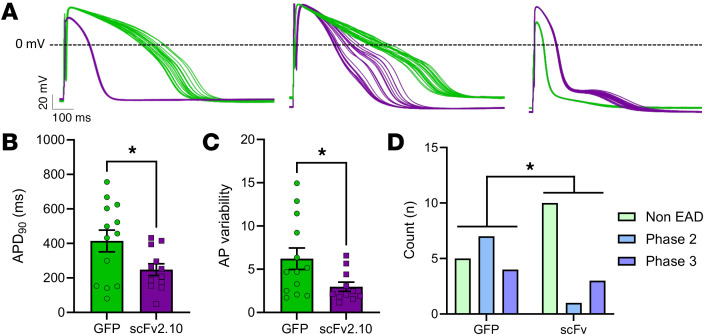
scFv2.10 expression reduces markers of proarrhythmia in JLN hiPSC-CMs. (**A**) Representative AP recordings from JLN hiPSC-CMs transduced with GFP (green) or scFv2.10 (purple). Left, no-arrhythmia (No EAD); center, phase 2 EADs; right, phase 3 EADs. (**B** and **C**) APD_90_ and AP beat-to-beat variability calculated from AP recordings as shown in A. *P* values were determined by unpaired, 2-tailed Student’s *t* test. Data are presented as mean ± SEM. (**D**) Distribution of GFP-transduced and scFv2.10-transduced JLN hiPSC-CMs generating APs with EADS (Phase 2, blue; Phase 3, purple) or without EADs (green). The 2 groups were compared using a χ^2^ contingency test. *P < 0.05.

Our data demonstrate that disabling the hERG1a PAS domain with scFv2.10 increases I_Kr_. To test if scFv2.10-mediated PAS disruption mitigates impaired repolarization in the absence of I_Ks_, we measured APs at 1 Hz pacing from JLN hiPSC-CMs transduced with either scFv2.10 or GFP (Figure 7A and Table 3). We measured 3 markers of proarrhythmia: APD_90_, beat-to-beat APD variability, and the incidence of EADs. beat-to-beat APD variability is a stronger predictor of arrhythmogenic potential than APD alone (31), and EADs are a cellular manifestation of cardiac arrhythmia (32). scFv2.10 transduction shortened APD_90_ by approximately 200 ms and reduced both Beat-to-beat APD variability and the incidence of EADs compared with GFP-transduced controls (Figure 7, B–D). These data demonstrate that disabling the PAS domain with scFv2.10 is antiarrhythmic in JLN hiPSC-CMs and suggest that targeting the hERG1a PAS domain could be a viable therapeutic target to treat impaired cardiac repolarization.

## Discussion

The present study investigated the antiarrhythmic potential of disrupting the hERG1a PAS domain by overexpressing the PAS-targeting hERG1 activator scFv2.10 (19). We demonstrated that scFv2.10 selectively enhances I_Kr_ magnitude, shortens APD, and reduces the incidence of EADs and beat-to-beat APD variability in JLN hiPSC-CMs. These data highlight the antiarrhythmic potential of targeting the hERG1a PAS domain.

### The hERG1a PAS domain as a more selective therapeutic target.

Though it is well-established that targeted enhancement of I_Kr_ shortens APD, hERG1 activators can display off-target and/or proarrhythmic effects in models of disrupted cardiac repolarization (33, 34). In a rabbit model of LQTS type 1, NS1643 shortened APD and the QT interval but also increased ventricular fibrillation. NS1643 contains a pharmacophore that nonselectively activates BK and K_2P_ channels (3, 7, 33). NS3623, a structural analog of NS1643, impaired conduction velocity and prolonged the QRS interval in guinea pig hearts (34). ICA-105574 reduced arrhythmic events in guinea pig hearts but increased susceptibility for arrhythmia at higher concentrations (33). Similarly mallotoxin, a naturally occurring hERG1 activator, shortens APD but promotes ventricular fibrillation in isolated rabbit hearts (35, 36). These data highlight the shortcomings of current hERG1 agonists and the need to develop new, selective hERG1 activators.

PAS domains typically detect stimuli and regulate function across numerous physiological processes. They can sense changes in redox potential or light exposure and play a role in circadian rhythm and ion channel gating (for a detailed review of PAS domain function, see ref. 37). PAS domains are typically activated by binding to cofactors such as ions or nucleotides (38–41); however, there are no reported hERG1 PAS-targeting ligands or small-molecule modulators. To our knowledge, we are the first to show that a PAS-targeting hERG1 activator reduces arrhythmic susceptibility in a LQTS background.

scFv2.10 selectively binds the hERG1 PAS domain to disable PAS domain activity. scFv2.10 should, therefore, minimize the potential off-target effects seen with other hERG1 activators that bind more classical hERG1 activator binding sites (7, 42). However, there are potential challenges that could arise with an scFv therapeutic. Fast clearance times due to its small size and immunogenic responses that degrade the antibody fragment would both reduce its efficacy in correcting abnormal cardiac repolarization (43). Sustained hERG1 activation could also overcorrect for QT prolongation and be proarrhythmic (6). Although there are pertinent risk factors regarding the application of a scFv, our work highlights the therapeutic potential of targeting the hERG1 PAS domain.

### Nuclear ion channel immunofluorescence.

Multiple ion channel subdomains are targeted to the nuclei of cells, including channels encoded by *CACNA1A* (44), *CACNA1C* (45), and *TRPM7* (46, 47). Most recently, our lab demonstrated that the distal C-terminal domain of hERG1 is targeted to the nuclei of immature cardiomyocytes; from the nucleus, the hERG1 nuclear polypeptide regulates ion channel function (27). In this study, we serendipitously identified and validated a KCNQ1-derived nuclear immunofluorescence signal. The mechanisms by which this KCNQ1 nuclear fragment is generated and targeted to the nucleus are beyond the scope of this study; however, the continued identification of nuclear-targeted ion channel subdomains may suggest that nuclear-targeted subdomains are be a common regulatory mechanism of ion channel activity. In past studies, nuclear ion channel fragments were shown to regulate gene expression, altering not only ion channel function (27, 44, 45) but also embryonic development (44, 46, 47). Further work is needed to determine the contribution of the KCNQ1 nuclear fragment in cell function and broader aspects of human physiology.

### Differential scFv activity.

The original characterization of scFv2.10 in HEK293 cells stably expressing hERG1a demonstrated that scFv2.10 destabilized hERG1 inactivation and accelerated the time course of deactivation at room temperature (19). The same study reported in hiPSC-CMs that scFv2.10 increased steady-state current density but did not measure effects on I_Kr_ gating kinetics. A follow-up study in HEK293 cells showed that scFv2.10 also accelerated hERG1a deactivation at 37°C (30).

In our hands, lentiviral scFv2.10 expression accelerated deactivation in HEK293 cells at room temperature but slowed the deactivation time course of I_Kr_ in JLN hiPSC-CMs. The increase in hERG1a to hERG1b subunit abundance likely explains the slowed gating observed in this study, but the mechanism by which this shift occurs is unclear.

It should be acknowledged that further work could be done to corroborate the reported shifts in hERG1 subunit abundance. This study only used immunocytochemistry to quantify the effects of scFv2.10, and experiments that more directly measure subunit abundance, such as Western blot, would bolster this finding.

The differences observed on native I_Kr_ between this study and previous work using scFv2.10 may be attributed to our lentiviral scFv2.10 expression in lieu of acute intracellular delivery through the recording pipette (19). The sustained scFv2.10 expression could affect hERG1 subunit assembly. hERG1b contains an endoplasmic reticulum retention signal in its N-terminus that is masked by the hERG1a N-terminus. In the absence of hERG1a, the majority of hERG1b subunits are retained in the endoplasmic reticulum and degraded (48, 49). Chronic scFv2.10 expression may alter hERG1a/1b subunit interactions, exposing the hERG1b retention signal and thereby selectively promoting hERG1a homomeric channels. It is also plausible that scFv2.10 binding of the nascent hERG1a N-terminus disrupts hERG1a/1b heteromerization at the ribosome, further promoting hERG1b retention and degradation. scFv2.10 delivery through the patch clamp recording pipette only effects assembled hERG1a/1b channels at the surface membrane and does not affect hERG1 channel assembly or forward trafficking within such a short exposure window.

scFv2.10 may also be acting as a chaperone. Multiple hERG1 channel blockers, such as E-4031 or pilsicainide, act as chemical chaperones by stabilizing hERG1 protein folding to promote hERG1 at the surface membrane (50, 51). This stabilizing effect is only observed with chronic drug treatment, whereas acute treatment has no effect (50, 51). scFv2.10 may act in a similar manner, but through targeting the N-terminal domain rather than the pore domain like most hERG1 blockers.

Multiple reports have reported that hERG1 expression is functionally linked with KCNQ1 expression (52, 53). KCNQ1 disruption has been reported to diminish hERG1 expression (29, 53, 54). Consistent with these reports, we found that the hERG1 immunofluorescence signal in JLN hiPSC-CMs was nearly half that of df19.911 hiPSC-CMs. Interestingly, scFv2.10 expression enhanced I_Kr_ magnitude to levels comparable with df19.911 hiPSC-CMs. In JLN hiPSC-CMs, scFv2.10 may act as a substitute chaperone for KCNQ1 to promote hERG1 expression. Another possibility is that scFv2.10 may stabilize hERG1a channels at the surface membrane by acting as a scaffolding protein. We must acknowledge that the 2 cell lines are not isogenic, and thus, these differences could be the consequence of genetic variability between individuals. We could not generate an isogenic control due to the genetic complexity of our JLN patient-specific line. Future studies could explore this phenomenon using gene-edited cardiomyocytes from an isogenic background.

Finally, while this work demonstrates the therapeutic potential of targeting the hERG1a PAS domain, hiPSC-CMs do not reproduce all facets of the adult myocardium (55). Many of our cells displayed spontaneous activity, a prominent marker of immaturity, despite maturation on a PDMS substrate (28, 55). Furthermore, recording I_Kr_ and I_Ks_ from hiPSC-CMs is challenging due to their small currents. Thus, it is possible there are kinetic changes we are unable to resolve in our patch-clamp recordings. Regardless, our data highlight an approach for hERG1 activation to treat cardiac electrical disorders that can be broadly applied.

## Methods

### Sex as a biological variable.

Both of the hiPSC lines used in this study, df19.9.11 and JLN, were derived from male individuals. Although LQTS displays sex-specific effects (56), the differential effects of scFv2.10 on cardiac repolarization in male and female tissue is beyond the scope of this study.

### HEK293 cell culture.

We maintained cells at 37°C and 5% CO_2_ in a Heracell incubator (Thermo Fisher Scientific). We cultured HEK293 cells in minimum essential medium (MEM, Invitrogen, 11095080) supplemented with 10% fetal bovine serum (Thermo Fisher Scientific, SH30070.03), and split cells once they reached 80% confluency.

### hiPSC line generation.

Somatic reprogramming was used to generate iPSC lines from skin fibroblasts from the patient with JLN carrying KCNQ1 W188X/Exon 3 deletion by expression of the reprogramming factors SOX2, OCT4, NANOG, LIN28, KLF4, SV40LT, and c-MYC, using nonintegrating episomal vectors (26). We generated, karyotyped, characterized, and cryopreserved 3 iPSC clones (Cellular Dynamics International).

### Stem cell culture and cardiac differentiation.

We used 2 human iPSC lines for this study; the df19.9.11 iPSC line served as a healthy control (WiCell) and the JLN hiPSC line was derived from a patient with JLN syndrome. As previously described, human iPSC were cultured and differentiated into cardiomyocytes using the GiWi protocol (28), in house or by the University of Michigan Cardiovascular Regeneration Core Laboratory. Briefly, we seeded stem cells on Matrigel-coated plastic plates in iPS-brew medium (Miltenyi Biotec). We checked cells daily to remove spontaneous differentiation and passed at 80% confluence. For cardiac-directed differentiation, ~80,000 cells were plated into each well of a 6-well plate and cultured to ~100% confluency. Cells were first treated with GSK3 inhibitor (day 0) to induce mesodermal differentiation, followed by a Wnt inhibitor (day 2) to induce formation of the cardiac mesoderm. On day 4, the Wnt inhibitor was removed to direct cells into cardiac progenitor cells. After 8–10 days, cardiac monolayers began spontaneously contracting. The monolayers were cultured to day 20 and subsequently purified using the human iPS-derived cardiomyocyte isolation kit (Miltenyi Biotec), following the manufacturer’s protocol. Purified cardiomyocytes were plated as a monolayer into a Matrigel-coated polydimethylsiloxane (PDMS) 6-well plate (~200,000 cells/well) for 7 days and then trypsinized and replated as single cells on Matrigel-coated PDMS (~8,000 cells/well). Patch-clamping experiments were completed at least 7 days after replating.

### Lentiviral constructs and transduction.

Lentiviral particles were produced using the lentiviral generating plasmid pLentiLox 3.7 (pLL 3.7), which contains a U6 promoter for shRNA expression and a CMV promoter for GFP expression downstream of the U6-siRNA expression cassette. scFv2.10-GFP was subcloned into the pLL 3.7 *Nhe*I-*Bsr*GI restriction sites. Empty pLL 3.7 vectors expressing GFP were used as controls. Lentiviral constructs and particles used in this study were generated by and purchased from the University of Michigan’s Vector Core. Cells were transduced at a multiplicity of infection (MOI) of 100. Transduction efficiency was assessed by observing GFP fluorescence 72 hours after transduction.

### Lentiviral production and determination of lentiviral titer unit.

HEK293 cells were grown to 50% confluency and transfected with a pLL 3.7 plasmid. Medium was changed 6 hours after transfection and incubated for 48–72 hours at 37°C. Viral supernatant was harvested 48–72 hours after transfection and stored at –80°C. HEK293 cells were transduced with 1× lentivirus, and fluorescence was analyzed at 72 hours. Titer was calculated as follows:


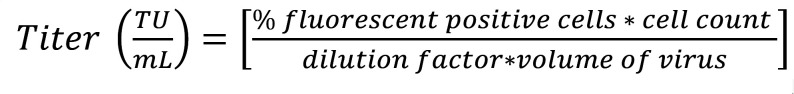


### Immunocytochemistry.

hiPSC-CMs were seeded on Matrigel-coated PDMS and fixed with 4% paraformaldehyde/PBS for 15 minutes, before being washed for 5 minutes with PBS followed by incubation with blocking buffer 21. (PBS [Gibco] + 1% BSA [Alfa Aesar] + 0.5% Triton X [IBI Scientific] + 10% Goat Serum [Vector Laboratories]) for 1 hour. Cells were incubated with primary antibodies in blocking buffer overnight at 4°C. The next day, cells were washed with PBS (5 minutes, 3 times) and incubated with secondary antibodies in blocking buffer for 1 hour at room temperature in the dark. Cells were washed with PBS (5 minutes, 3 times) and mounted with ProLong Gold antifade reagent (Thermo Fisher Scientific) on a coverslip.

hiPSC-CMs were immunolabeled for hERG1a (ALX-215-050-R100, Enzo Life Sciences), hERG1b (ALX-215-051-R100, Enzo Life Sciences), or hERG1 p-loop (ALX-804-652-R300, Enzo Life Sciences) using a 1:200 dilution of the primary antibodies followed by a 1:250 dilution of secondary antibody goat anti–rabbit Alexa Fluor 647 (4050-31, Southern Biotech). KCNQ1 was labeled using a 1:100 dilution of the primary antibody (ab84819, Abcam) followed by a 1:250 dilution of secondary antibody goat anti–mouse Alex Fluor 568 (A11004, Invitrogen). Actin filaments were labeled using phalloidin (A12379, Thermo Fisher Scientific) to confirm the presence of sarcomere structure. Nuclei were labeled using 1:1,000 dilution of DAPI (1 μg/mL) for 15 minutes (2248, Thermo Fisher Scientific). Immunostained preparations were analyzed using a confocal microscope (Zeiss 880) to determine protein expression.

### Electrophysiology.

All ionic currents (I_Kr_, I_Ks_, I_Ca_) were recorded at physiological temperature (37°C ± 1°C) using whole-cell patch clamp with an IPA Integrated Patch Amplifier run by SutterPatch (Sutter Instrument) and Igor Pro 8 (Wavemetrics).Borosilicate glass recording pipettes (2–5 MΩ) were backfilled with intracellular solution containing: 5 mM NaCl, 150 mM KCl, 2 mM CaCl2, 5 mM EGTA, 10 mM HEPES, and 5 mM MgATP, adjusted to pH 7.2 with KOH; all chemicals were purchased from Sigma-Aldrich, unless otherwise stated. Cells were perfused at a rate of ~2 mL/min with extracellular solution that consisted of 150 mM NaCl, 5.4 mM KCl, 1.8 mM CaCl_2_, 1 mM MgCl_2_, 15 mM glucose, 10 mM HEPES, and 1 mM Na-pyruvate, pH 7.4 adjusted with NaOH. I_Kr_ and I_Ks_ were reported as an E-4031 (2 μM) and chromanol-293b (50 μM) sensitive currents, respectively. Series resistance for whole-cell recordings ranged from 3 to 15 MΩ. A 100 ms step to –50 mV was applied prior to I_Kr_ and I_Ks_ recordings to inactivate sodium currents. Voltage was then stepped to a 3-second pulse from –50 to +50 mV in 10 mV increments followed by a 10 second pulse at –40 mV. Data were sampled at 5 kHz and low-pass filtered at 1 kHz. Steady-state I_Kr_ and I_Ks_ density was calculated as the 5 ms mean at the end of each prepulse normalized to capacitance as a function of prepulse potential. I_Ca_ density was calculated as the peak I_Ca_ normalized to capacitance as a function of prepulse potential. Tail I_Kr_ density was calculated as peak tail I_Kr_ normalized to capacitance as a function of prepulse potential and fitted with the following Boltzmann equation:


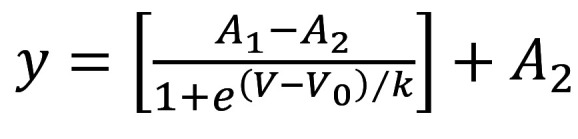


where A_1_ and A_2_ represent the maximum and minimums of the fit, respectively, V is the membrane potential, V_0_ is the midpoint, and k is the slope factor. The time course of I_Kr_ deactivation was calculated by fitting current decay during the 3-second pulse at +20 mV with a double exponential function:


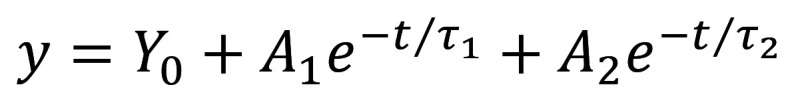


where Y_0_ is the asymptote, A_1_ and A_2_ are the relative components of the fast and slow time constants *t*_1_ and *t*_2_, respectively.

We recorded action potentials at physiological temperatures (37°C ± 1°C) using a perforated patch with amphotericin B (0.3 mg/mL) in current clamp mode, as previously described (57). Borosilicate glass recording pipettes (2–5MΩ) were backfilled with intracellular solution supplemented with amphotericin B. Cardiomyocytes were paced at 1 Hz following perforation, which was observed as a rapid hyperpolarization of the resting membrane potential that stabilized within 60 seconds. Series resistances for perforated patch AP recordings were between 22 and 100 MΩ. The time to APD_90_ and APD_90_ variability were calculated from the average of 20 successive paced APs within a cell. Cells that could not be paced at 1 Hz were not included in AP analyses.

### Statistics.

Data were analyzed using IgorPro and GraphPad Prism. We evaluated data for normality (D’Agostino-Pearson and Shapiro Wilk tests) and outliers (ROUT test) before statistical evaluation in GraphPad Prism. Data were also considered outliers if they fell outside the average ± 2 times the SD. APD and deactivation data were compared using a parametric (normal distribution) or nonparametric (nonnormal distribution) 2-tailed Student’s *t* test. We ran the Mann-Whitney *U* or Kolmogorov-Smirnov nonparametric test when groups showed similar or unequal variances, respectively. We considered variance unequal if there was a 2-fold or greater change in the SD or 95% CI. Steady-state and tail I_Kr_ and I_Ca_ were analyzed using 2-way ANOVA (mixed methods) with a Šidák post hoc test. I_Ca_ recordings with a calculated voltage error > 6 were excluded from analysis. Only I_Kr_ and I_Ks_ subtractions yielding positive current densities are reported. We compared the count of EADs between GFP-transduced and scFv2.10-transduced cells using a χ^2^ contingency test. Statistical significance was taken at *P* < 0.05.

### Study approval.

Written informed consent was obtained from the patient in accordance with the last version of the Declaration of Helsinki and with approval by the University of Wisconsin Health Sciences institutional review board.

### Data availability.

All data are stored in perpetuity in the Deep Blue Data repository at the university of Michigan and are available upon request. Supporting data values are also provided in the supplement (supplemental material available online with this article; https://doi.org/10.1172/jci.insight.183444DS1).

## Author contributions

CUU, ENJV, LLE, TJK, and DKJ wrote the manuscript. CUU, SS, MG, FGSC, KMO, AJ, ENJV, and DKJ completed experiments. LLE and TJK generated the patient-derived stem cell line. CUU, SS, MG, FGSC, KMO, AJ, ENJV, and DKJ completed data analysis. CUU and DKJ conceived the study.

## Supplementary Material

Supporting data values

## Figures and Tables

**Table 1:**
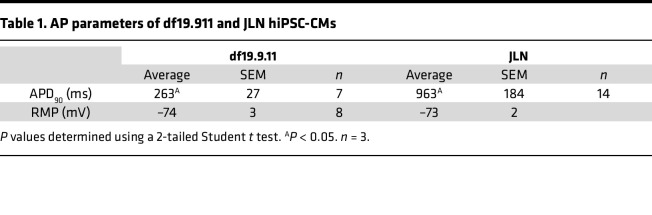
AP parameters of df19.911 and JLN hiPSC-CMs

**Table 2:**
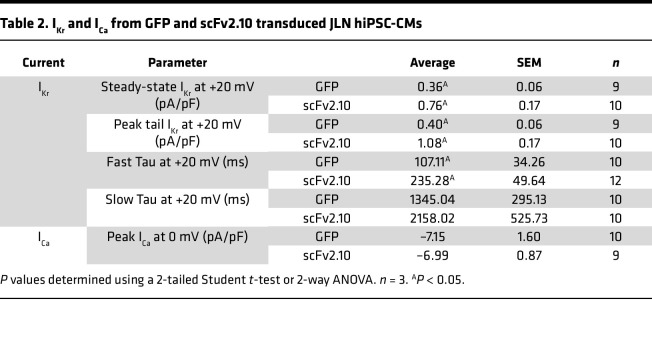
I_Kr_ and I_Ca_ from GFP and scFv2.10 transduced JLN hiPSC-CMs

**Table 3:**
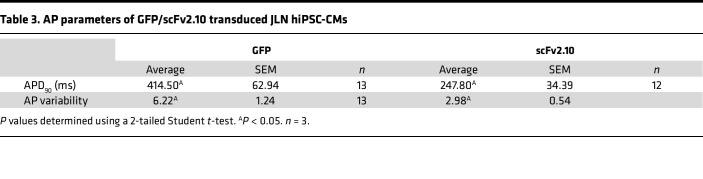
AP parameters of GFP/scFv2.10 transduced JLN hiPSC-CMs

## Version 1 01/09/2025

In-Press Preview

## Version 2 02/24/2025

Electronic publication
